# Fear and anxiety: Affects, emotions and care practices in the memory clinic

**DOI:** 10.1177/0306312718820965

**Published:** 2018-12-25

**Authors:** Julia Swallow, Alexandra Hillman

**Affiliations:** School of Sociology and Social Policy, University of Leeds, Leeds, UK; School of Social Sciences, Cardiff University, Cardiff, UK

**Keywords:** affect, Alzheimer’s disease, diagnosis, emotion, memory clinics

## Abstract

This paper contributes to the growing recognition in Science and Technology Studies and medical sociology of the significant role of affect in scientific and clinical work. We show how feelings of fear and anxiety associated with dementia not only shape people’s experiences and responses to a diagnosis, but also shape the practices and processes through which assessments and diagnoses are accomplished. What emerges from our research, and provides a distinct contribution to this growing field of study, is the relationship between the uncertainties that pervade the diagnosis of memory problems and the various strategies and practices employed to care for, divert, restrict or manage affective relations. Furthermore, our ethnographic material illustrates the implications of this relationship: on the one hand, it provides opportunities for care work through ‘tinkering’ with diagnostic technologies and extending and opening out diagnostic categories, while on the other, it can form part of healthcare practitioners’ disposal work, restricting opportunities for alternative meanings of dementia to endure.

## Introduction

… they [patients] can be angry … they can be frightened, anxious. (Observation Memory Nurse, describing his patients’ responses to cognitive assessment)

This nurse’s description of the memory clinic patients he meets is illustrative of the kinds of emotionally charged encounters that make up the everyday work of UK memory clinics. Memory clinics are places in which people, mainly older people, and their relatives seek explanations for a wide variety of troublesome experiences, most commonly related to a perceived worsening in cognitive function, including memory. The processes of assessment, consultation and diagnosis that ensue are shaped by the anxieties and fears associated with Alzheimer’s disease (AD) or other causes of dementia that have come to represent a threat to ones very soul (Halewood, 2016). This article takes into account the feelings and emotions that characterize memory clinic work to illustrate the kinds of affective relations produced through the negotiation and experience of emotions, and to reflect on their implications for clinical protocols, practice, and patients’ and families’ experiences of assessment and diagnosis.

Diagnosing Alzheimer’s disease is a complex and uncertain process, not least because of the difficulty in classifying symptoms associated with both ‘normal’ and pathological ageing processes but also because of the worry and anticipation that it causes for patients and their families. AD is often metaphorically conceived as a ‘fate worse than death’ (Zeilig, 2012), or even a living death (Behuniak, 2011), due to its association with a gradual decline of independence and agency, resulting in a complete loss of self (Beard, 2016). These images have a direct impact on the clinical consultation and yet exactly how these affective responses of fear and anxiety are entangled in the clinical encounter and in the accomplishment of diagnoses requires critical examination. Drawing on two ethnographies of memory clinics in the UK, we illustrate the important relationship between these affective economies (Ahmed, 2004a), and contribute to a growing literature within Science and Technology Studies (STS) and the sociology of medicine, that captures the ways in which uncertainty, and the tools and strategies employed to tackle it, can be utilised to provide a means with which to attend to and care for affective relations, or to traverse, contain or defer them.

## Background

### Affect and the affective turn in STS

An important starting point for our interpretations of the assessments and diagnostic practices that happen in the memory clinic is to acknowledge and make visible the role of affect in the production and accomplishment of clinical facts and their communication. We are mindful that the term ‘affect’ carries with it significant and increasingly contentious theoretical debate across a range of literatures spanning the humanities and social sciences (e.g. Leys, 2011; also the response by Frank and Wilson, 2012). We also recognize that some theorists call for sharp distinctions to be made between different forms of response, such as emotion and affect (Massumi, 1995). Drawing on feminist theories of affect (see Pedwell and Whitehead, 2012), and the work of Ahmed (2004a, 2004b) in particular, we do not seek to privilege the term affect but instead to recognize how feelings such as fear and anxiety, which are associated with dementia, are negotiated and experienced in memory clinic encounters. Part of this recognition is to reflect upon both the relations of power and the politics of selfhood that are imbued in these experiences and practices. In doing so, we illustrate that affects surpass the binaries of embodied emotions on the one hand and meaning, discourse and politics on the other.

Our paper builds upon the significant attention within STS on the affective entanglements in the clinic and laboratory which shape the production of scientific and clinical knowledge (see Fitzgerald, 2013; Kerr and Garforth, 2015; Lynch, 1985; Pickersgill, 2013; Wetherell, 2012). According to Kerr and Garforth (2015), we have seen an ‘affective turn’ in STS where ‘embodiment, care and affective interactions’ are the focus of studies examining the intellectual and epistemic work of science (e.g. Fitzgerald, 2013; Myers, 2008; Pickersgill, 2012). We also aim to develop work in STS which recognizes the ways in which care, with the different forms of attachments and alignments it encompasses (De la Bellacasa, 2011), shapes and configures the production of scientific knowledge and medical practice, or in our case the assessments and diagnosis of dementia, both ethically and epistemologically (see Pickersgill, 2012).

### Affect and care in scientific and clinical work

Our study of memory clinic work attends specifically to the relationship between affect, and material and technical practices of care. De la Bellacasa (2011, 2012, 2015) invites us to consider how caring labours constitute scientific thinking and knowing; extending analysis to the affective practices through which scientific practices are performed and accomplished. As we show through our ethnographic material, care and affect are not mutually exclusive; rather, as Giraud and Hollin (2016) note, ‘the value of care lies in its capacity to combat instrumentalization through creating the space to affect and be affected’ (p. 4). De la Bellacasa (2011) frames ‘care’ as an ‘affective state’ with the ability to ‘re-affect objectified worlds’ (p. 89). Building on Latour’s (2004) conception of ‘matters of concern’, De la Bellacasa (2011) introduces the idea of ‘matters of care’ stressing that a critical approach is crucial, since the world is unequal and increasingly stratified. For Puig de la Bellacasa, care as an affective state operates in recognition of stratification and marginalisation and cannot therefore be reduced to a universal set of principles, which defines how to care ‘well’; care is continually at work and emergent in practice across different sites, such as the memory clinic.

For Mol et al. (2010), the technical dimensions of care work are constituted with respect to the ‘tinkering’ of socio-technical infrastructures to negotiate the ambivalences and tensions inherent to world-making: care is ‘attentive to suffering and pain, but it does not dream up a world without lack … care seeks to lighten what is heavy, and even if it fails it keeps on trying’ (p. 14). We also draw on Mol et al.’s (2010) use of the idea of tinkering with respect to the role of technologies in the memory clinic. Here, technologies are not in and of themselves careless (i.e. ‘bad’) but also require tinkering and adapting to specific situations as part of the wider practices of care in the clinic. There is therefore a politics of care that requires interrogation (c.f. Tronto, 1994).

Feminist ethics of care shows not only that ‘care gets things done, but that more or better or different care could be generative of better survival, politics, and knowledge’ (Martin et al., 2015: 628). Feminist visions of care emphasize it as political, messy and dirty; it is a ‘non-innocent’ category (De la Bellacasa, 2012; Haraway, 2012; Murphy, 2015; Tronto, 1994) and despite being an ‘everyday doing’ it can also become and reinforce systemic regimes of power (see Ticktin, 2011). Martin et al. (2015) critically engage with ‘care’s darker side’ by drawing attention to its complexity beyond ‘a romantic or laudatory treatment of the theme’ (p. 627). In doing so, they address its power dynamics since it has the ability to organize, classify and discipline bodies. Recognizing this darker side of care is integral to understanding the relations between care practices and affective relations in the memory clinic, where attaching a diagnosis of dementia can be a means with which to order and discipline ageing, forgetting bodies. As theorized by scholars across STS, the material and technical dimensions of care work are relational and context-specific (see Barad, 2007; Haraway, 1991; Silverman, 2012) and situated in practice, action and a ‘willingness to respond’ (Martin et al., 2015: 634).

### Care, uncertainty and productive affects

Alongside efforts to examine affect and emotion in the production of medical work, recent scholarly attention has also elucidated the role of uncertainty in the production of clinical knowledge and practices of medical work (see Leem, 2016; Moreira et al., 2011; Street, 2011). Uncertainty, with respect to the production of biomedical knowledge, can be utilized as a ‘valuable resource’ rather than a problematic hindrance to the expertise and epistemology of diagnosis and disease categorization (see Moreira et al., 2011; Street, 2011). We develop this thesis to explore the potential utility and resource offered by the uncertainty which surrounds a diagnosis of AD, not only for accomplishing processes of assessment and diagnosis in the memory clinic, but also for negotiating feelings and emotions and thus shaping the affective economy of dementia diagnosis. We also argue, however, that the practice of negotiating affects and emotions in the memory clinic has the potential to engender new uncertainties, (re)producing the affective economies of fear and anxiety as they circulate and ‘stick’ in the clinic (Ahmed, 2004a). Ahmed (2004a, 2004b) focusses on emotions as analytical tools, arguing that ‘emotions do things’, moving between bodies and sticking figures together. Fear in connection with the diagnosed AD patient does not reside within particular bodies or figures; rather, the anticipated future with AD works to stick this fear.

Through fear not only is the very border between self and other affected, but the relation between the objects feared (rather than simply the relation between the subject and its objects) is shaped by histories that “stick”, by making some objects more than others seem fearsome. (Ahmed, 2004a: 128)

Although in her discussion of fear Ahmed (2004a) exclusively discusses racism, terrorism and migration as points for analysis, we develop her conceptual work to explore how fear and anxiety of dementia have stuck, passed through into the clinic. We consider how these affective economies are subsequently negotiated between healthcare practitioners, patients and their families. Through various forms of care work in the diagnostic process, alongside the hesitations, extensions and reflections that the uncertainty of diagnosing AD offers, healthcare practitioners are able to utilize, rather than dispose of, this fear and anxiety.

We show how various forms of care work provide spaces in which a ‘willingness to respond’ is opened up, demonstrated by the tinkering of cognitive screening tools in the clinic (Mol et al., 2010) and the slowing down of the diagnosis process (De la Bellacasa, 2015). Here, time is made through ‘socio-technical arrangements and everyday practices’ (De la Bellacasa, 2015: 694), as practitioners work to extend the time taken for diagnosis in an effort to mediate feelings of fear or worry. Yet, simultaneously, these technical and material care practices can work to discipline and order bodies in the clinic in order to manage the inherent uncertainties of diagnosis. Practices of assessment are subsequently shaped both by the organization and routinization of clinical work to discipline ageing, forgetting bodies through diagnosis, *and* the ‘response-ability’ (Haraway, 1991; Viseu, 2015) of healthcare practitioners to attend to feelings of fear and anxiety as ‘matters of care’.

## Methods

This article draws on two ethnographies of memory clinics. The two studies included fieldwork in four memory clinics in the UK, one in South West England, one in South Wales and two in Yorkshire. Swallow’s ethnography, an ESRC-funded doctoral study on ‘The role of instruments for screening cognitive function and Alzheimer’s disease’, included observations in multi-disciplinary team meetings with clinical professionals working across the fields of psychiatry and psychology and in initial consultations with healthcare practitioners, patients and family members where cognitive screening tools were used. Interviews were carried out with a number of clinical professionals. Hillman’s ethnography, funded through a Wellcome Trust postdoctoral fellowship award, was carried out in two large university teaching hospitals, one in an urban and the other in a rural area. The study involved observations of clinical consultations as well as interviews with clinic staff, patients and family members.

All clinics functioned in similar ways, assessing patients experiencing problems with thinking and memory. The most common route through which patients attended the memory clinics was through referral from their general practitioner (GPs). Other routes included referral from another community or primary care service such as day centres, or less commonly referral from secondary services, relatives, carers or the patients themselves. Following referral, patients have an initial assessment which involves cognitive tests, the taking of a detailed patient history by asking questions of the patient themselves and a relative/carer, and clinical tests including blood tests, a trace of the heart – if it is a possibility that the patient may require medication for their memory which carries contraindications for some heart arrhythmias – and, increasingly, a Computerized Tomography (CT) scan of the brain. In Swallow’s study the initial appointments were mainly carried out by memory nurses, either in the patient’s home or in clinic. For Hillman’s study, the memory nurses – as well as student psychologists – carried out the cognitive tests during initial appointments, but it was more commonly the consultant old-age psychiatrist or geriatricians who took overall responsibility for the initial assessment, incorporating the results of the cognitive test. In all sites, the cognitive testing makes up (at most) 25 minutes of the clinical encounter, unless the patient is having difficulty understanding or answering the questions. At this point, the practitioner can allocate more time depending on overall caseload. The tools of pertinence to this article are the Addenbrooke’s Cognitive Examination (ACE 111) and the Montreal Cognitive Assessment (MoCA).

The fieldwork across all clinics was made up of observations in the memory clinics. This involved both the audio recording of clinical consultations, alongside observations and the taking of fieldnotes of the encounters. This approach captured the talk and interactions involved in initial patient assessments, the discussion of test results and processes of diagnoses, as well as the broader social, material and spatial contexts in which these encounters took place. Over the periods of observation across all four clinics, 61 consultations were observed. As well as the in-clinic observation, the researchers interviewed 36 memory clinic staff, 21 patients who had attended a memory clinic 19 relatives/carers (ten of the patients and relatives were interviewed twice and one couple were interviewed three times) and ten research experts working in the field of dementia.

In both studies, memory clinics emerged as spaces made up of interactions between different kinds of healthcare practitioners (nurses, doctors, psychologists and psychiatrists for example), and between practitioners and patients and their families. Diagnosis is arrived at through the ‘assemblage’ and ‘juxtaposition’ (Latimer, 2013) of clinical tests, cognitive tests, interpretation of patient histories and a diagnosis by exclusion (Bender, 2003).

In the following analysis, we focus initially on how practitioners engage in tinkering practices to adapt or mold cognitive screening tools in response to the fear and anxiety exhibited by patients and family members. We then show that beyond the role of technologies, practitioners also extend points of uncertainty and slow down the diagnostic process. Drawing on De la Bellacasa’s (2015) work on the pace of care, we show how time, as enacted through the slowing down of diagnosis, is crucial to both the production of clinical knowledge in relation to AD and to the management of affective relations. Fear and anxiety are also deferred, displaced, contained and restricted as practitioners deal with the uncertainty of the medical decision-making process. We argue that these practices are performed as a way of seeking or establishing certainty and to accomplish patient disposals (c.f. Berg, 1992) where in the routinization and organization of memory clinic work, a diagnosis of dementia may be a means through which to discipline and order bodies. Here we address the recent call in STS to ‘stay with the trouble’ (Martin et al., 2015; Murphy, 2015). We conclude by reflecting on the fact that uncertainties and anxieties produced by the diagnosis process are utilized as opposed to disposed: Just as fear and anxiety ‘stick’, so do various forms of care work in everyday practice, undertaken as necessary strategies for making sense of AD for patients and family members.

## Tinkering practices, technologies and relations of care

I’m not, I’m not, it’s not a threatening thing you know at the end of this test, it doesn’t you’re not gonna say ‘oh well right because of this test you’ve got Alzheimer’s’. You know you can’t do that so it’s, it’s making ‘em feel ‘I’ll have a go at doing this you know I’m not under pressure it doesn’t matter if I get it wrong nobody’s going to laugh at me, nobody’s going to criticize me’ you know and if they get it wrong I don’t point that out. I don’t say ‘ooh you’ve got that wrong’ you know they just literally go through it bit by bit and then after, I always say ‘well done’ whatever they’ve done you know, ‘this is lovely now thank you’. (Memory Nurse)

As this extract illustrates, in order to negotiate the uncertainties of accomplishing a diagnosis of AD and the accompanying feelings of fear and anxiety, practitioners engaged in tinkering practices (Mol et al., 2010). Tinkering became necessary for dealing with uncertainty and for navigating and mobilizing the networks of practices involved in the medical decision-making process. We reflect on such tinkering as engendering relations of care between technologies, practitioners, patients and their families, as a means for making sense of uncertainty in the clinic and to account for the *affects* produced by the consultation process. In doing so, we show how these practices utilize and value uncertainty.

As the memory nurse quoted above describes, the cognitive screening tools are mediated through practitioners’ tinkering to negotiate the emotions that circulate in the clinic. To account for and make sense of fear and anxiety entangled with diagnoses, the memory nurse also reassures patients and in doing so performs a great deal of emotional labour throughout appointments. This memory nurse also performs this work to negotiate the uncertainties inherent in the technologies themselves, which, as she explains, cannot be used to fix a diagnosis. Such reassurance was central to a number of consultations we witnessed. Practitioners would continually encourage patients, stopping to say *‘well done’*, telling them to just *‘do their best’*, and therefore producing moments of care. Such tinkering practices are crucial for making sense of uncertainty and for negotiating the emotions, which pass through into the clinic.

The following extract from an interview with a clinical psychologist confirms that the process of classifying AD overall is complex,

It is often a process of exclusion rather than confirmation in terms of the diagnostic process for dementia so I think it’s dangerous if we attach too much importance to the tests. (Clinical Psychologist)

Here, conceiving the tools as partial or incomplete systems to metaphorically ‘box’ AD reflects the uncertainty associated with AD more broadly; classification is a process of exclusion, rather than confirmation. For this clinical psychologist, it is important not to privilege any one technique or technology, given the uncertainty in classifying what AD is (Jutel, 2011). This does not mean that the tools are rendered redundant in the classification process. In fact, the everyday and routine practices of the clinic bring them ‘to life’ (Berg, 1996: 501), shaping how cognitive decline and AD are measured and how complexity is taken care of.

The negative affects associated with these tools are felt by both the person being assessed as well as the professional administering the test; responses to these affects are therefore not necessarily wholly benevolent responses to another’s fear, but may also reflect professional and organizational concerns and interests. As we will see further in the paper, these everyday routines and habits of the memory clinic are also useful strategies through which to manage and traverse affective relations, demonstrating both how practices of assessment are shaped by the organization of clinical work and the ‘response-ability’ (Haraway, 1991) of practitioners to attend to uncertainty and feelings of fear as ‘matters of care’.

Alongside the complexity associated with determining what AD *is* through the use of particular technologies, the disease remains stigmatised and feared (Beard, 2016; Beard and Neary, 2013). As the following excerpts from interviews carried out with practitioners shows, this fear of the abject, ‘senile other’, dominates patients’ concerns. Across the memory clinics, the ‘fear’ of AD passes through into the clinic and sticks, creating further uncertainty for practitioners to manage (c.f. Ahmed, 2004a). Emotions, as described by Ahmed (2004a), are aligned with particular constructions of the future, in this case constructions of futures with possible AD. As one clinical psychologist explains, the ‘horror’ of Alzheimer’s persists.

I think people don’t understand it and I think people think about it in terms of being mental I saw a chap before you came in and he said, ‘am I mental’? You know and of course that’s not what I’m looking at ever really so I think there’s a lack of there’s just a general lack of understanding I think, people’s experiences of dementia is usually of their older relatives who were treated quite poorly. (Clinical Psychiatrist)

Here, the horrors associated with AD, including the ‘otherness’ and the ‘social death’ marked by institutionalization (see Taylor, 2010), are concerns for patients experiencing memory problems. This was also a point of reflection for this consultant psychiatrist,

There can be a tendency to minimize symptoms – that’s either conscious or a subconscious one – because of the fear you know, ‘you’re going to put me in a home, you’re going to send me off to some institution somewhere or other’. (Clinical Psychiatrist)

Decline in cognitive function marks a process of becoming, which enacts visions of a ‘loss of self’ to the ‘horrors’ of institutional care, a narrative that pervades individuals’ expectations regarding cognitive decline (see Taylor, 2010). Here ‘fear as an emotional reaction’ to the futurity of diagnosis and institutional care was witnessed across clinical consultations (Ahmed, 2004a). Emotions circulate in the clinic, which leads to some patients masking symptoms and resisting diagnosis. Practitioners continually negotiate the vulnerabilities of the initial consultation with awareness of the anxieties and anticipations entangled in diagnosis.

In what follows, we show that tinkering practices in the clinic are performed in response to both the difficulties in categorizing AD and the uncertainties and anxieties concerning the meaning of diagnosis for patients and their families. We illustrate the many ways in which practitioners carefully choreograph the consultation process. They are able to ‘take care’ of the vulnerabilities of the diagnostic encounter and manage fears inherent in diagnoses. They die so through practical crafting of the technologies – for example, missing questions on the test – and through practitioners’ emotional labour as they ‘reassured’ patients throughout the process.

Tinkering forms a part of practitioners’ practical manipulation to ensure that cognitive decline becomes a ‘manageable problem’ for both practitioner and patient (Berg, 1996: 504). Practitioners manipulate the tests in a number of practical ways, including changing the order of the tests and actively omitting sections of the tests. As Swallow (2016) has shown, such tinkering work (re)constitutes the tools as provisional devices inscribed with ad hoc procedures for the purpose of transforming and making sense of complexity. Rather than this manipulation work rendering the tools useless (Moreira, 2010) such work is performed to negotiate the complexities associated with diagnosis, for making patients feel reassured, to ensure they ‘do their best’ (Consultation Memory Nurse) (p. 129). Yet, in ‘taking care’ of the vulnerabilities of the diagnostic encounter as emotions circulated in the clinic, it could be argued that such manipulation and mediation work was performed to enable staff to manage these negative affects to ensure an ordered process of assessment. Here we capture the politics of care work; the practices of ‘taking care’ are the ‘duties of the powerful’ seen here with respect to the management of negative affects to discipline the assessment process (Tronto, 1994: 114).

## The pacing of dementia diagnosis

The problem, you know, unfortunately we don’t have an easy test that you can measure and say, ‘You are definitely on the early stages of Alzheimer’s Disease because your blood test shows this and that and the other.’ We don’t have any of that. So we really have to try to rule out any other possibilities or any other possible causes for the problem. Sometimes it’s a matter of seeing you over a period of time and see how things go and then we’ll be more – we’ll have more idea, a better idea on what the possible cause for this is. So, nothing definite I can tell you at the moment, but the only thing definite I can tell you is that yes, you have done well in some of the tests, but some others, like the memory, the speed of retrieving information is a bit slow, the concentration is a bit slow as well. (Memory clinic doctor)

As we have shown, actors tinker to utilize uncertainty in the interactional processes of the clinical encounter, shaping their affective relations. In this section, we show how practitioners further demonstrate a ‘willingness to respond’ through the pacing of a dementia diagnosis to utilize uncertainty (Martin et al., 2015: 634). Practitioners can allow for moments of uncertainty to linger, making time for diagnosis – often as a response to a perceived risk of emotional distress. The extract above comes from an interview with one of the doctors working in the memory clinic. She describes a common approach to the mediation of fears and anxieties; this was echoed by a number of practitioners working in the clinics.

In this doctor’s description, we are once again able to see the uncertainties and challenges of reaching a definitive diagnosis of Alzheimer’s disease and other causes of dementia. Even at the point of attaching a clinical label, many doctors describe it as a ‘working’ diagnosis, something that is always in process, being re-assessed and worked at. Time is described by the doctor as a means through which to reach a better sense of what the cause of the problem might be. Time and slowing down the pace of diagnosis, as well as the slowing down and spreading out of the disease itself, is shown to be a central component through which practitioners were able to mediate feelings of fear or worry in the everyday encounters in the memory clinic, as this next extract exemplifies.

Mrs D: Can slow …

VR: … slow down the progression of things. So that can – yes, make you feel a bit better, yes. Not a cure, not a cure, but it could improve the symptoms.

BD: Slow, slow down.

VR: Exactly, yes, yes. Okay. Good. (Memory clinic doctor)

In this exchange, one of the doctors explains to their patient that medication can slow down progression of the disease. Careful to highlight that the medication is not a cure, she focusses on keeping deterioration at bay. The patient responds saying ‘slow, slow down’ reiterating the temporal benefits of the drug and becoming enrolled in the spreading out of the diagnosis through a delaying of its impacts and implications. This extension of time, opening out processes of assessment and re-assessment and extending the meanings of the diagnosis may represent an example of De la Bellacasa’s (2015) care time, which ‘suspends the future and distends the present, thickening it with a myriad of demanding attachments’ (p. 707). Many practitioners spoke of time as a means through which they were able to build better relationships with patients and families, allowing them to be more responsive to the contextual specificities of their problems. For example, the extract below from one of the memory clinic nurses describes the reaching of a diagnosis in terms of the changing nature of family relationships and the time required for them to come to terms with these changes.

You know, for relatives it’s the change in dynamics in the relationship for, you know … especially these days, lots of the patients have been the ones that were picking the grandchildren up from school and the ones that were depended on greatly and then suddenly these people are now becoming dependant again. (Memory clinic nurse)

This thickening of time creates a space in which practitioners have opportunities to attach value to and care for the multiple and often competing set of concerns that shape dementia and its diagnosis. Yet this care time exists in tension with a more ‘urgent, speedy temporality’ (De la Bellacasa, 2015), that attends to the increasing demands on memory clinics to diagnose more people more quickly. This competing temporality compresses the present and restricts the potential for practitioners to attend to diagnosis as a matter of care (De la Bellacasa, 2011), as this memory clinic doctor describes:

But I guess now at the moment we have the pressure to really diagnose within two visits because we don’t have capacity to be able to do it anymore. (Memory clinic doctor)

## Displacement, deferral and traversing fear and anxiety: Accomplishing clinical disposals

But usually I tell the carer first when the patient’s away and I’ll say look we’ve done all this, we’ve put – and I guess you have stock phrases that I’m sure the psychologist sitting with me is a bit fed of hearing – but stock phrases of ‘I guess my job here is to put all the pieces of the puzzle together and we’re putting the testing and the scan and there’s no one thing that’s telling me this but I think you’re right to have brought these concerns ‘cause I’m worried as well that there is something more serious going on, have you any thoughts what you think it could be?’ (Memory clinic doctor)

In the case of diagnosing AD, routines enable practitioners to recognize, account for and to some extent overcome the complexity and uncertainty that characterizes the diagnosis of AD. According to Berg (1992: 170), these routines are performed with a ‘certain “automatism”: habitually, without explicitly reflecting on or legitimating the actions involved’, something we identified in the interactions we observed in the memory clinics. Even in the case of communicating a diagnosis, the words and phrases can become habitual or routinized, as the doctor in the extract above illustrates. In the communication of diagnostic assessment, the doctor describes how they draw on a stockpile of resources to make sense of and negotiate the complexity associated with diagnosis. The habitual performance of routines during assessments and consultations highlight the politics of care within the memory clinic, whereby there is a tension between attending to feelings of fear and anxiety, on the one hand, and strategies of simplification, on the other.

We have demonstrated that uncertainty offers opportunities for care enacted through the tinkering of technologies and the pacing of diagnosis. However, given the complexity and uncertainty surrounding the diagnosis of dementia, as well as the presence and circulation of affective relations, the question remains as to how medical decisions are accomplished. This section of our analysis therefore focusses on how practitioners deal with ambiguities and uncertainties in their work. In particular, we show how accomplishing patient disposals (Berg, 1992) can result in practices to defer, displace and restrict affect and emotion. In order to maintain legitimacy and authority over the diagnostic act, practitioners must manage through routines what Berg (1992: 169) has referred to as the ‘utter chaos’ of medical problem-solving.

Drawing on the everyday routines of clinical work, practitioners are able to maintain the definition of the situation (Goffman, 1978) and choreograph ‘appropriate’ displays of affective response. In this sense, deferral work with respect to the articulation of a diagnosis in the clinic extends the constraining and containing of emotional responses to diagnosis within ‘acceptable’ boundaries of a clinical encounter, as the following extract illustrates:

You know and they did want a diagnosis but after you’ve given them that diagnosis in the clinic, they then go home and then they sit and they think they’re you know they’re literally devastated by it. (Trainee Psychiatrist 1)

In this case, the desire to know the diagnosis in the clinic and the displayed devastation of knowing it once at home, is highlighted as a point of contrast. Similarly, the extract below from a nurse practitioner explains the lack of reaction to a diagnosis displayed in the clinic as either a reflection of a lack of insight on the patient’s part or simply demonstrative of a lack of effect of the diagnosis.

Very often I will say to people the most likely explanation is something like Alzheimer’s disease, I’m sure you’ve heard about that and very often it goes straight over their heads, or there’s no discernible catastrophic effect … you can use that word and the person isn’t going to collapse in a heap on that floor.

(Memory clinic nurse)

We suggest that this described phenomenon, in which the diagnosis has less impact than is feared, or in which there is less reaction than is expected, may be a reflection of the carefully managed ‘definition of the situation’ (Goffman, 1978) of the clinical encounter. Here, displays of emotion are constrained and feelings are contained to accomplish a diagnosis, at least while they are within the routines and practices of the clinic. This produces a tension between moments of care work and performance of routines as they serve to accomplish diagnoses in the clinic through a closing down of displays of emotion. We therefore demonstrate the tension between practitioners’ ‘willingness to respond’ (Martin et al., 2015) and their efforts to manage what is a clinically ‘appropriate response’ to diagnosis. Whilst practitioners allow for moments of uncertainty to linger, this can also result in efforts to manage the appropriateness of a response to emotional distress and ‘close down’ emotions in the clinic, as the following extract highlights.

I: Yeah. And do you think people are perhaps a bit more comfortable at home?

R: Yeah, everybody’s more relaxed. I remember when we first started doing that, because as I say our roles have sort of extended, you know, got wider over the years I’ve been here. I remember the first time that I gave somebody the diagnosis at home and the chap had quite a lot of insight and him and his wife were just in floods of tears. But if they were in the clinic, the probably wouldn’t have been able to do that, you know, and it was just nice that they could do that ‘cause that was like a natural reaction. And I had the time to be with them. I’m really upset … it’s stupid.

I: Sorry.

R: Sorry, it’s awful when you talk about it.

Outward reactions to a diagnosis are shaped by socio-material environments and the meanings attached to settings and situations that define what an ‘appropriate’ response is. In this extract, the practice nurse becomes emotional and tearful herself, reflecting her engagement in what she describes as a ‘natural’ reaction to hearing the diagnosis. According to the nurse, the couple were ‘able’ to show their feelings at home in a way that they would ‘not be able to’ in the clinic. Our purpose in exploring these different perspectives on patients’ reactions to hearing the diagnosis is not to make judgements regarding the appropriate level of emotional response, or to suggest that the clinic should be made more like a home, but rather to show the connections between the careful work practitioners do to manage the definition of the situation (utilizing the mundane and routine practices of clinical work) and affective relations, whilst also accomplishing patient disposals. As well as drawing on uncertainty in the diagnostic process, routines and mundane practices of clinical work can also be utilized to manage and traverse the presence of fear and anxiety and perhaps to protect practitioners from ‘too much’ emotion.

Finally, as we illustrated in the first section of our analysis, uncertainty and ambiguity can be employed, rather than contained, in the memory clinic. However, unlike the examples we explored previously, there are moments in which the utility of uncertainty can operate as a mechanism through which to traverse and contain affective relations resulting in the *intensification* of fear and anxiety, as this extract from fieldnotes taken during a consultation illustrates:

Mrs Jones and her daughter come to the clinic to discuss the difficulties that Mrs Jones is experiencing with her speech. Her ability to communicate verbally has become increasingly difficult, to the extent that she often relies on writing notes to communicate, carrying a small notebook with her everywhere she goes. After Mrs Jones’ daughter describes the problems her mum is experiencing, while her mother undertakes some cognitive tests in the room next door, the doctor responds:

I think, just as far as the labels are concerned, you were saying, was it dementia or was it this, that or the other, I don’t think …. There are bits of her brain that aren’t working properly; dementia just means some loss of some mental abilities. Well, on that basis she’s got some loss, so definitely she’s got some form of dementia. The problem is, people mean, immediately you say dementia, they interpret it differently and they assume that it’s inevitably progressive and that people go ga-ga when their memory’s a problem.

Well, actually that’s just because conditions like Alzheimer’s disease probably typically causes memory problems. So yes, she has some cause of dementia, but probably it’s not a particularly helpful word to use because it pushes the wrong buttons.

In this case, the uncertainties of dementia and Alzheimer’s disease are made a central part of its communication. The labels are challenged and opened up. This allows those present to attend to feelings of fear, embodied in the figure of dementia. Emotions in this context, as Ahmed (2004a) asserts, are aligned with wider socio-historical landscapes, in this case the conceptions of dementia as signifying the threat of progressive loss of self, accompanied by the stigmatization of madness and senility. In this specific clinical encounter, we see that emotions mediate diagnostic processes – not just between the individual actors, but between actors and the wider collective. Meanings of dementia are therefore integral to both the production and the mediation of fears. The doctor’s response attends to two kinds of fears: the present fear of what this deterioration in Mrs Jones’ speech might mean, and the anticipated fears produced through the circulating meanings of dementia as a signifier of loss and decline.

When Mrs Jones arrives back in the consultation room, the doctor asks her if it is important to her to know what the cause of her speech problem might be.

Doctor: Again, is it a concern to you what might be the cause of the speech problems?

Mrs Jones: Yes.

Doctor: Okay. Well …

Mrs Jones: I get very frustrated.

Doctor: I can understand, yeah. I think it’s part of the brain isn’t working properly and it will be a part of the brain over here and that’s probably because the brain cells in that part of the brain just gradually are not working. It’s a little like people who develop Alzheimer’s Disease. In Alzheimer’s Disease it’s the memory part of the brain that doesn’t work. In your case it’s the talking part of the brain. Okay? So it’s not anything to worry about or be concerned about particularly.

Mrs Jones: It’s easy for you to say.

Here we see again the breaking down of categories, speaking instead about the specificity of what is happening in the brain, only making limited connections to a diagnostic label, in this case explaining that it is ‘a bit like Alzheimer’s disease’. The extent to which this is successful in displacing feelings of fear is unclear. Efforts to restrict connections to dementia to displace feelings of fear may work to (re)produce fear and anxiety. In disciplining patients’ concerns and anxieties this encounter may instead intensify emotions as a result of attempts by the practitioner to reorder the chaos of the clinical encounter. Mrs Jones’ response that ‘it’s easy for you to say’ suggests that it remains a worry and a concern for her, even with the attempts to break up diagnostic labels and challenge their meanings. This attempt to defer fear and anxiety, by removing labels viewed to be stigmatizing, may actually result in an intensification of feelings by obscuring or detaching the object of fear to one that has less material or temporal presence (c.f. Ahmed, 2004a), something to which we return in our discussion.

## Discussion

In this article, we illustrate affective economies of the memory clinic, capturing the connections between uncertainty, affect and the everyday accomplishment of clinical decision-making in the diagnosis of dementia. The uncertainties that characterize AD diagnosis become implicated in the production of affective relations. As we have shown, this relationship is both responsive to the fear and anxiety engendered as a result of the stigma associated with AD and the abject future that a diagnosis of AD carries, and is productive of care practices and strategies to adapt and utilize uncertainty in clinical work. We also highlight an important duality in the implications of uncertainty for affective relations. On the one hand, uncertainty provides opportunities for care work such as tinkering in the carrying out of cognitive tests and the slowing down and extending of diagnosis, providing opportunities for alterity and attachments. On the other hand, we show how strategies for coping with uncertainty, through the routinization of clinical work, can result in practices to contain affect for the accomplishment of patient disposals. This can restrict the possibilities for multiple meanings of memory loss to coexist. For example, as seems to be the case for Mrs Jones, this has the potential to intensify feelings of fear and anxiety amongst patients and families. By attending to this duality, we demonstrate how care works affectively.

We began our article by exploring how emphasizing uncertainty may be understood as a form of caring practice, opening up opportunities for tinkering and the utilization of care time, demonstrating practitioners’ ‘willingness to respond’ (Martin et al., 2015). Extending moments of uncertainty and holding on to aspects of collectivity in processes of assessment and diagnosis might provide the resources to produce ethical (or careful) practice (see Kerr et al., 2007 for a similar argument regarding ambiguity in relation to the use of genetic research). Prolonging such moments – as opposed to shutting down uncertainty and condensing memory loss into the individual’s body (Moreira, 2010) – affords practitioners a greater degree of sensitivity to the relatedness of patients’ own experience of memory loss and the social practices that inform and sustain personhood. This was reflected in the nurse’s account of the importance of allowing time in the diagnostic process for patients and families to come to terms with the changing dynamics of family relationships. The multiple decisions regarding the communication of test results and diagnoses can potentially become enrolled in a more continual process of taking care of patients, families and community relations. Our work therefore suggests a different approach to thinking about the implications of uncertainty in clinical work. Uncertainty can often be seen to heighten patients’ and families’ worries and fears about whether what they are currently experiencing is indicative of a worse fate to come. What our work shows is how this uncertainty can provide the means with which to recognize and care for (through tinkering or the pacing of care) the affective economies, as Ahmed (2004a) would describe them, associated with the circulating meanings of dementia.

We are also careful to ‘stay with the trouble’; we recognize and illustrate the darker side of care, through which healthcare practitioners rely on simplifications and routinization to order and discipline ‘emotional’ bodies, to solve medical problems and accomplish a diagnosis with a degree of authority and legitimacy. Our work therefore also attends to the mundane, organizational routines of clinical assessments and diagnosis. These practices are shown to not only aid practitioners in accomplishing patient disposals (Berg, 1992) in the face of extreme complexity and uncertainty, but are also shown to be a means with which practitioners are able to traverse the affective economies of dementia diagnosis, protecting themselves from ‘too much’ emotion while simultaneously maintaining the work of memory services with increasing numbers of patient referrals. These practices can include a reliance on the maintenance of clinical routines, utilizing the habits and rituals of everyday clinical work to sustain the continuation of memory clinic work alongside the feelings of anxiety, fear and horror that are associated with the diagnosis of dementia. Such care practices are not performed by practitioners solely as an act of benevolence but may reflect professional concerns as well as organizational efforts to reach diagnostic closure.

Finally, we show how affective relations and uncertainty are connected through the temporal frame with which dementia diagnosis is accomplished. Uncertainty provides the potential for a slower, extended temporality in which dementia diagnosis becomes a matter of care. This ‘care time’ may provide the possibilities for attachments to be made to multiple meanings of dementia that help and enable people to live with and alongside dementia (Latimer, 2013). However, we have also illustrated that part of the means through which practitioners try to manage and displace feelings of fear is through a utilization of uncertainty that relocates the object of fear, as a distant and potential future entity, which, as Ahmed (2004a) suggests, can intensify fear through its anticipation.

These ethnographic insights into practices of assessment and diagnosis in UK memory clinics provide a means of conceptualizing the utility of uncertainty in clinic work: a resource for accomplishing diagnosis and negotiating affective relations. In demonstrating this relationship between uncertainty and affective relations, we make visible some of the previously hidden aspects of dementia diagnosis and their implications. We show that this relationship can provide opportunities for care work that may engender ‘a willingness to respond’ (Martin et al., 2015) with sensitivity to the meanings that circulate memory clinics about dementia and Alzheimer’s disease. Affective relations are traversed or constrained in a variety of circumstances, but in part as a result of the mundane strategies employed to maintain the accomplishment of clinical decision-making and diagnosis.
